# Primary antiphospholipid syndrome presenting with unilateral renal infarction and C4d-positive cortical necrosis: a case report with a pooled analysis of 24 cases

**DOI:** 10.3389/fimmu.2025.1713159

**Published:** 2026-01-12

**Authors:** Jing Zhang, Lina Zhang, Sheng-Guang Li, Ji Li, Yadan Zou, Ting Long, Ruohan Yu, Yanfeng Zhang

**Affiliations:** Department of Rheumatology and Immunology, Peking University International Hospital, Beijing, China

**Keywords:** anticardiolipin antibody, anticoagulation, antiphospholipid syndrome, C4d, complement activation, cortical necrosis, lupus anticoagulant, renal infarction

## Abstract

Primary antiphospholipid syndrome (APS) is an autoimmune thrombophilic disorder characterized by recurrent venous or arterial thrombosis and/or pregnancy morbidity in the presence of persistent antiphospholipid antibodies (aPL). Renal involvement in APS is heterogeneous and may include renal artery thrombosis, renal vein thrombosis, thrombotic microangiopathy, and chronic APS nephropathy. However, unilateral renal infarction as the first and isolated manifestation of primary APS is exceedingly rare. We report a 23-year-old man with no prior medical history who presented with fever, flank pain, and hematuria and was found to have unilateral left renal infarction with cortical necrosis. Imaging showed a slender mid-to-distal segmental defect in the left renal artery with markedly reduced perfusion and extensive cortical non-enhancement. Laboratory testing revealed persistent lupus anticoagulant (LA) and high-titer anticardiolipin antibody (aCL) IgG, fulfilling criteria for primary APS. A percutaneous renal biopsy, performed to clarify the mechanism of injury and exclude immune-complex glomerulonephritis or primary vasculitis, demonstrated diffuse cortical necrosis with fibrin-rich thrombi in small arteries and intense C4d deposition along glomerular capillary and small arterial walls, without immune-complex deposition. We also conducted a pooled analysis of 24 APS cases with renal infarction identified in the literature. Most involved arterial thrombosis, often in young patients, with variable outcomes depending on laterality and timeliness of anticoagulation. Our case underscores several key teaching points (1): APS should be considered in young patients with unexplained renal infarction (2); transient positivity of multiple autoantibodies (including dsDNA, ANCA, and anti-GBM) may be misleading and requires cautious interpretation with repeat testing (3); C4d deposition supports complement-mediated thrombosis as a pathophysiologic mechanism in APS nephropathy; and (4) early diagnosis and anticoagulation are crucial for a favorable outcome.

## Introduction

Antiphospholipid syndrome (APS) is an autoimmune disorder characterized by hypercoagulability and recurrent thrombosis in association with persistent antiphospholipid antibodies (aPL), including lupus anticoagulant (LA), anticardiolipin (aCL), and anti-β2-glycoprotein I antibodies. Classically, APS manifests with venous thromboses (such as deep vein thrombosis and pulmonary embolism) and arterial events (including stroke, myocardial infarction, and peripheral arterial thrombosis), as well as pregnancy morbidity. Renal involvement in APS can be diverse, ranging from renal vein thrombosis and thrombotic microangiopathy to large-vessel renal artery thrombosis and chronic APS nephropathy (1–3). Among these, isolated renal infarction as an initial arterial event in primary APS is particularly unusual and may be easily misattributed to other etiologies (4–6).

**Figure 1:**
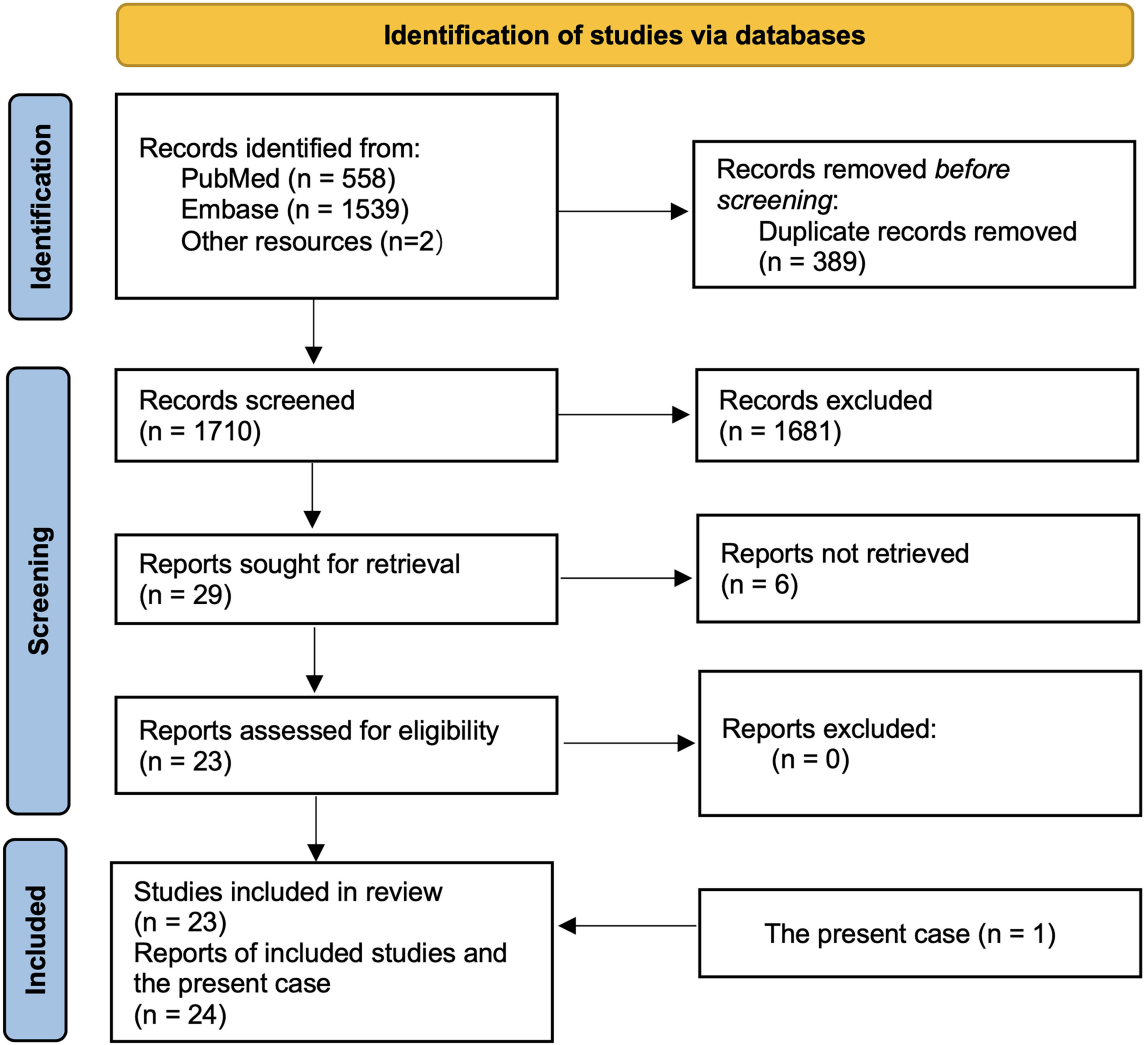
PRISMA-style flow diagram of the literature search and study selection. A systematic search was conducted on September 17, 2025, using PubMed, Embase, and additional sources. A total of 1,710 records were identified (PubMed n = 558, Embase n = 1,539, other sources n = 2). After removal of duplicate records (n = 389), 1,710 records were screened and 1,681 were excluded based on title and abstract. Twenty-nine reports were sought for full-text retrieval, of which six were not obtained. The remaining 23 reports were assessed for eligibility, and all were included in the qualitative synthesis. These 23 published studies, together with the present case (n = 1), yielded a final dataset of 24 cases included in the pooled analysis.

We present a case of primary APS in a young man who developed a unilateral left renal infarction with cortical necrosis. This case is, to our knowledge, among the first to document complement deposition in APS-related renal infarction. We also provide a literature-based overview of APS-associated renal infarction, discuss diagnostic challenges (including mimicry of SLE, AAV, or Goodpasture’s disease), and highlight management considerations. In addition, we conducted a structured literature search of reported APS cases with renal infarction using PubMed, Embase, and other sources. The search was performed on September 17, 2025, and covered all records indexed from database inception to the search date. A total of 1,710 records were identified (PubMed n = 558, Embase n = 1,539, other sources n = 2). After removal of duplicate records (n = 389) and title/abstract screening (n = 1,681 excluded), 29 reports were sought for full-text retrieval. Six reports could not be obtained, leaving 23 reports for eligibility assessment; none were excluded after full-text review. Thus, 23 published studies were included in the qualitative synthesis, and together with the present case (n = 1), constituted a final dataset of 24 patients for pooled analysis (**Figure 1**).

## Case presentation

A 23-year-old man with no past medical history presented with 14 days of left flank pain that began as a dull ache with colicky exacerbations and gross hematuria, accompanied by nausea and vomiting. Fever and chills developed on day 3 (maximum 40 °C). He denied lower urinary tract symptoms. At an outside hospital, laboratory tests showed WBC 15.37 × 10^9^/L, Hgb 161 g/L, platelets 91 × 10^9^/L, CRP 42.9 mg/L, urinalysis with numerous RBCs and 3+ protein, and serum creatinine 93.6 µmol/L. Abdominal ultrasonography showed an enlarged left kidney with absent cortical flow, suggesting left renal ischemia. He received LMWH 6000 U every 12 h plus empiric antibiotics; hematuria and proteinuria decreased, and urine color cleared, but fever persisted (peaking at 39 °C), prompting transfer to our center.

On admission to our hospital, he was comfortable with T 36.5 °C, BP 125/82 mmHg, HR 94 bpm, and SpO_2_ 99% on room air. Cardiac and pulmonary examinations were unremarkable. There was left costovertebral angle tenderness but no abdominal guarding or rebound tenderness. No skin rash, oral ulcers, Raynaud’s phenomenon, arthritis, lymphadenopathy, edema, or neurologic deficits were noted.

Laboratory tests now showed WBC 8.22 × 10^9^/L (neutrophils 69.3%), Hgb 149 g/L, platelets 326 × 10^9^/L, ESR 27 mm/h, CRP 31.2 mg/L, and procalcitonin 0.09 ng/mL (**Table 1**). Urinalysis revealed 1+ protein and +++ blood with approximately 22 RBCs/hpf; 24-h urine protein 0.46 g (slightly elevated). Creatinine was 109 µmol/L, BUN 5.67 mmol/L. Coagulation tests showed PT 11.3 s, APTT 39.2 s (slightly prolonged), and normal fibrinogen and D-dimer. Liver function tests and electrolytes were within reference ranges. Blood cultures were negative. Transthoracic echocardiography found no intracardiac thrombus or valvular vegetation; lower-extremity Doppler showed no DVT. Serologic testing for hepatitis B and C, human immunodeficiency virus, and syphilis was negative, and there was no clinical or laboratory evidence of systemic infection. The patient denied chest pain or dyspnea, oxygen saturation remained normal, and chest auscultation was unremarkable; therefore, a dedicated chest CT angiography for pulmonary embolism was not performed, and no cardiopulmonary thrombotic events occurred during follow-up.

**Table 1:** Admission laboratory and ancillary evaluations (key results).

Domain	Test	Result	Interpretation/note
Hematology & inflammation	WBC	8.22 ×10^9/L (Neut 69.3%)	Normalized
	Hemoglobin	117 g/L	Mild anemia
	Platelets	261 ×10^9/L	Recovered from initial thrombocytopenia
	CRP	2.91 mg/L	Improved
Coagulation	D-dimer	3165 ng/mL	Markedly elevated
Renal & urine	Creatinine	109 μmol/L	Stable renal function
	BUN	5.67 mmol/L	Within expected range
	Urinalysis	1+ protein; ~22 RBC/hpf	Persistent low-grade proteinuria & microscopic hematuria
	24-h protein	0.46 g/24 h	Slightly above normal
Autoimmunity (aPL & others)	Lupus anticoagulant	Positive	Meets APS lab criterion (persistent on repeat)
	aCL IgG	118.7 CU	High titer
	anti-β2GPI	IgG positive	Weak positive
	ANA	Negative	—
	anti-dsDNA	Positive	Transient; later negative
	Anti-nucleosome	Positive	Transient; later negative
	Anti-Scl-70	Positive	Transient; later negative
	MPO-ANCA	38 CU	Low-to-moderate **↑**; transient
	PR3-ANCA	23.4 CU	Weak positive; transient
	Anti-GBM	81.6 CU	Transient elevated; biopsy IF negative
	C3/C4	Within normal limits	No hypocomplementemia
	RF/anti-CCP	Negative	—
Microbiology	Blood/urine cultures	No growth	Infection unlikely
Embolic & thrombophilia work-up	Inherited thrombophilia panel	Not suggestive	Apart from aPL
	Transthoracic echocardiography	No thrombus/vegetations	Embolic source unlikely
	LE venous Doppler	No DVT	—

aCL, anticardiolipin antibody; aPL, antiphospholipid antibodies; anti-β2GPI, anti-β2-glycoprotein I; ANCA, anti-neutrophil cytoplasmic antibodies; BUN, blood urea nitrogen; CRP, C-reactive protein; DVT, deep venous thrombosis; IF, immunofluorescence; LE, lower extremity; MPO, myeloperoxidase; PR3, proteinase-3; RF, rheumatoid factor.

**Figure 2:**
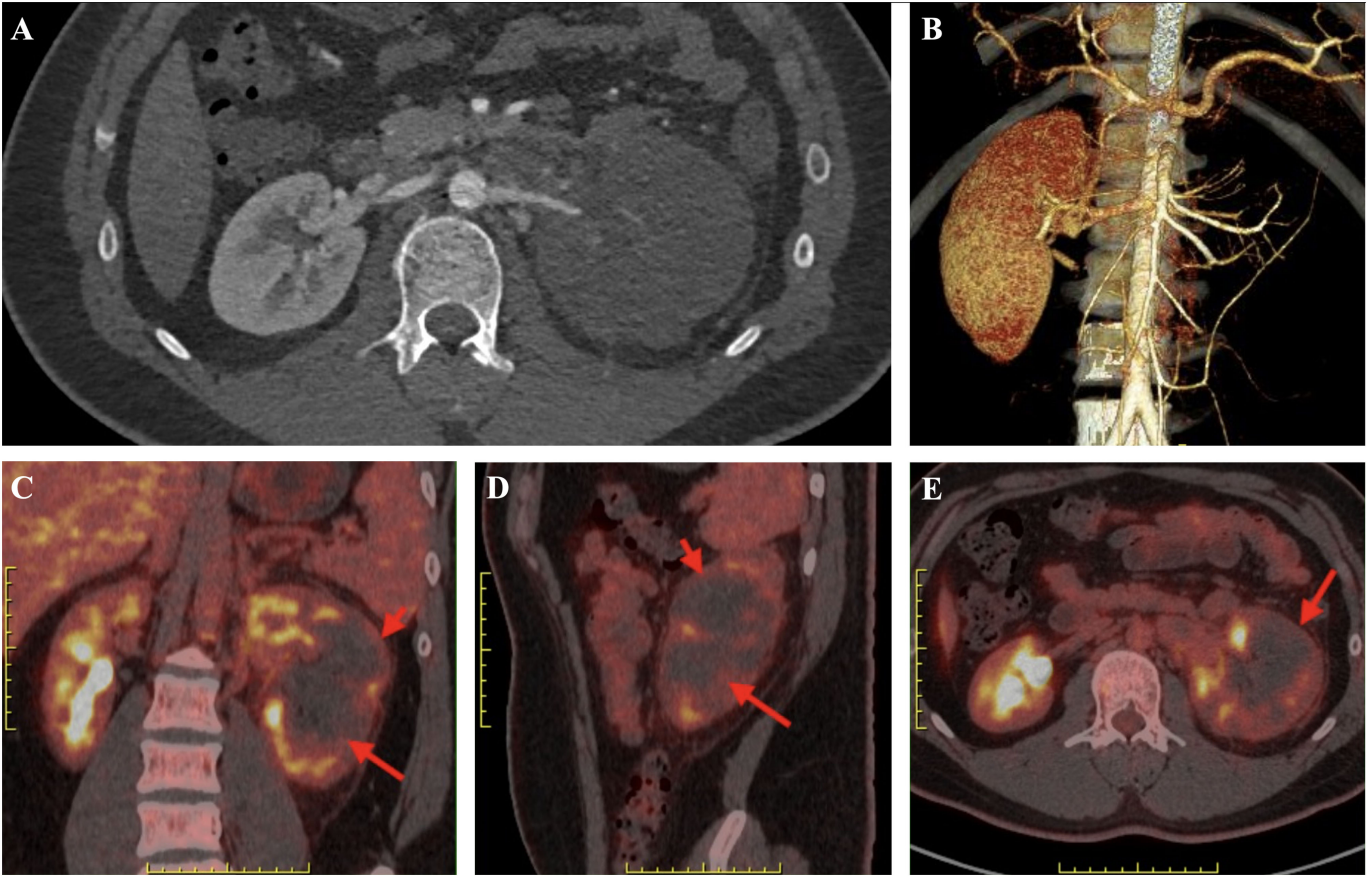
Imaging findings of unilateral left renal infarction with cortical necrosis. **(A)** Contrast-enhanced abdominal CT (axial) showing a markedly enlarged left kidney with loss of normal corticomedullary enhancement and a peripheral/patchy hypodense appearance consistent with large-area cortical infarction; the right kidney demonstrates preserved perfusion. **(B)** 3D CT angiographic reconstruction demonstrating markedly reduced arterial supply to the left renal parenchyma (attenuated/absent distal branches), compatible with major arterial occlusion or severe hypoperfusion of the left kidney. **(C–E)** Fused PET/CT images (coronal C, sagittal D, axial E). Red arrows denote photopenic regions corresponding to infarcted cortex with absent tracer uptake; yellow arrows indicate residual areas of mild tracer uptake likely reflecting reactive inflammation/viable parenchyma.

An abdominal CT angiography (CTA) demonstrated a slender mid-to-distal left renal artery with a segmental intraluminal filling defect and markedly diminished contrast opacification of the left kidney, consistent with arterial embolism/thrombosis (**Figures 2A, B**). The left renal vein appeared attenuated, raising concern for concomitant venous thrombosis. Delayed images showed extensive cortical non-enhancement and poor excretion, indicating cortical necrosis with extensive nonviable infarcted parenchyma (**Figures 2 C-E**). The right kidney was normal.

Autoimmune testing revealed ANA negative, but aCL-IgG 118.7 CU (high positive), aCL-IgM 33.8 CU (moderate positive), and lupus anticoagulant positive by dilute Russell’s viper venom time. Anti-β2-glycoprotein I antibodies were negative. Interestingly, several other autoantibodies were transiently positive at low to moderate titer: anti-double-stranded DNA (dsDNA), anti-nucleosome antibodies, p-ANCA with mildly elevated MPO-ANCA 38 CU and PR3-ANCA 23.4 CU, and anti-GBM 81.6 CU; C3/C4 were normal, and RF/anti-CCP negative. Twelve weeks later, repeat testing confirmed persistence of LA and high-titer aCL-IgG, while the transiently positive dsDNA, nucleosome, ANCA, and anti-GBM antibodies reverted to negative, and ANA remained negative.

To more precisely characterize the renal lesion and to exclude primary vasculitis or immune-complex glomerulonephritis in this young patient with atypical serologies, a percutaneous renal biopsy of the left kidney was performed on day 19 of illness after careful assessment of bleeding risk (normal platelet count and coagulation profile, and prior to initiation of long-term anticoagulation). The procedure was performed under ultrasound guidance by an experienced operator. The biopsy specimen was subsequently transferred to a collaborating external pathology institution for tissue processing and histopathologic evaluation.

Light microscopy demonstrated widespread ischemic necrosis of glomeruli and tubules (cortical necrosis), along with narrowed small arteries containing fibrin-rich thrombotic occlusions. Immunohistochemistry for C4d showed strong granular positivity along glomerular capillary loops and small arterial walls. No proliferative glomerulonephritis or immune-complex deposition was identified. These findings support ischemic infarction secondary to arterial thrombosis rather than lupus nephritis, ANCA-associated vasculitis, or anti-GBM disease.

### Management

With an arterial thrombosis plus persistent aPL, the patient met the revised Sydney criteria for definite APS. Anticoagulation was escalated to full-dose LMWH bridged to long-term warfarin (target INR 2.5–3.0). Given the extent of infarction and multi-autoantibody positivity, a short course of glucocorticoids (prednisone equivalent 0.8–1 mg/kg/day) was initiated, then gradually tapered. Hydroxychloroquine 0.2 g twice daily was added as adjunct. Aspirin 50 mg/day was introduced for arterial antithrombotic synergy. Fever abated and flank pain improved; platelets/coagulation normalized, and inflammatory indices fell; hepatic/renal panels remained stable. The patient was discharged on warfarin (INR-adjusted), prednisone tapered to 7.5 mg/day, hydroxychloroquine 0.2 g BID, and aspirin 50 mg/day.

### Follow-up

Over >2 years, the patient remained well with no recurrent thrombosis, hematuria, or systemic autoimmune manifestations. Serial laboratory tests showed stable renal function (creatinine 97–111 µmol/L), normal blood counts, and persistently negative ANA, dsDNA, nucleosome antibodies, ANCA, and anti-GBM antibodies. LA remained positive/weakly positive, and aCL-IgG remained high-positive, confirming persistent APS. Follow-up imaging revealed that the left kidney became smaller with scarring, and the right kidney compensated with preserved function. On serial testing, ANA remained negative, aCL-IgG remained high positive with weakly positive LA, and other transiently positive autoantibodies remained negative. He continues long-term warfarin with INR monitoring and multidisciplinary follow-up.

## Discussion

### Renal infarction in APS – rarity and mechanisms

This case highlights a rare presentation of primary APS: unilateral renal infarction with cortical necrosis as the first and isolated arterial event. Renal infarction is uncommon in general emergency practice and is typically associated with cardiac emboli (e.g., atrial fibrillation, mural thrombus), renal artery dissection, trauma, or hypercoagulable states. APS-related renal infarction has been described in only a limited number of cases and may involve large- or medium-sized renal arteries or intrarenal branches. Mechanistically, APS-related renal involvement can reflect large-vessel arterial thrombosis, renal vein thrombosis, or small-vessel thrombotic microangiopathy. In our patient, imaging showed a focal left renal artery filling defect with downstream cortical necrosis. Histology confirmed thrombotic occlusion of small arteries and arterioles with cortical necrosis, rather than an inflammatory vasculitis or immune-complex glomerulonephritis, strengthening the evidence for complement-mediated thrombosis in APS.

### Literature review of reported cases

**Figure 3:**
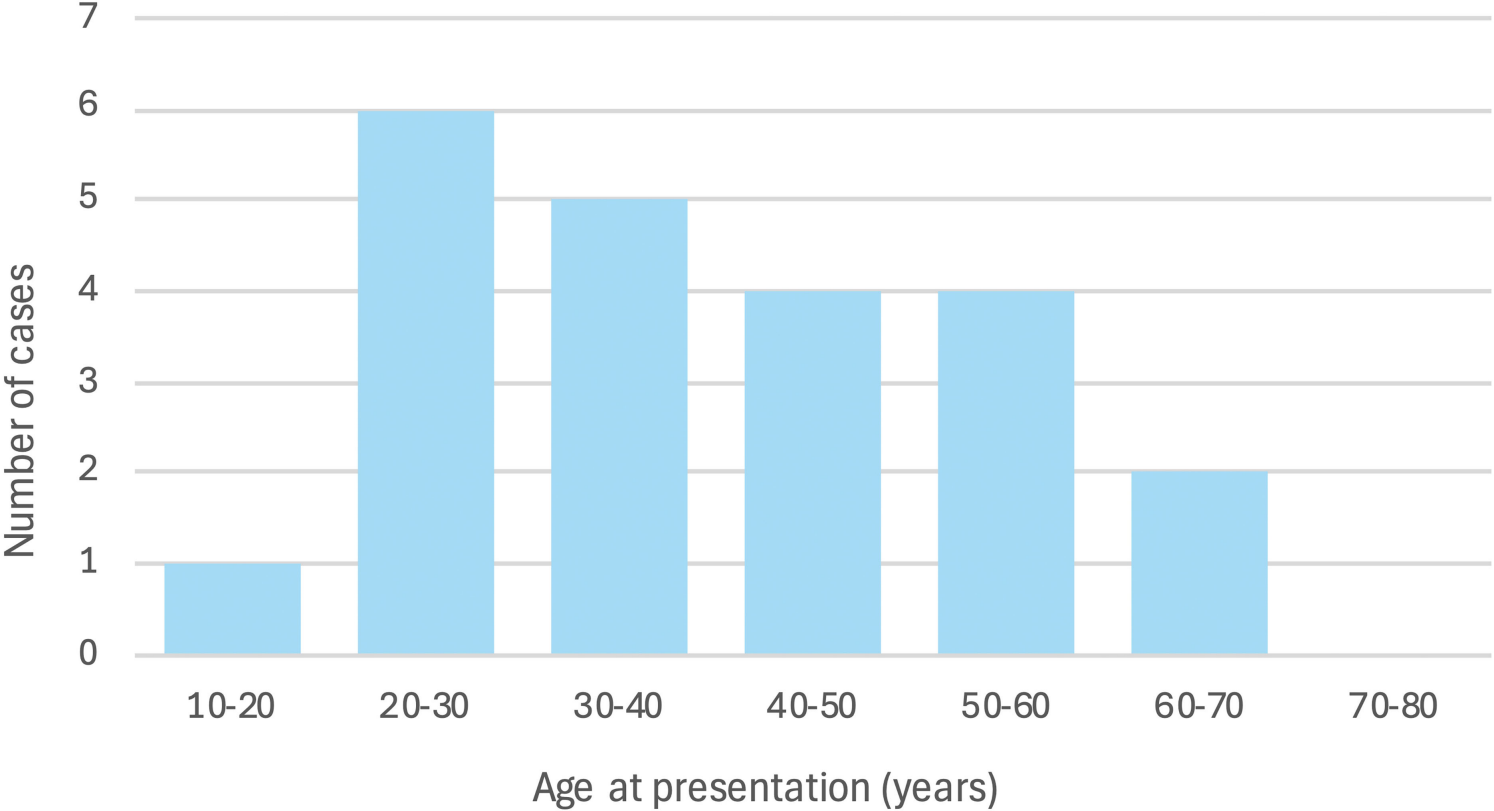
Age distribution of 24 APS patients with renal infarction (23 literature cases from **Table 1** plus the present case).

We performed a literature search of APS cases with renal infarction using PubMed, EMbase, and other sources, without date restriction, combining terms such as “antiphospholipid syndrome,” “renal infarction,” “renal artery thrombosis,” and “renal cortical necrosis”. Case reports and small series describing renal infarction in definite APS (primary or secondary) were included, while reports without clear APS criteria or non-English reports were excluded. After screening, we identified 23 published APS cases with renal infarction that met our predefined eligibility criteria. When combined with the present case (**Table 2**), the pooled cohort consisted of 24 patients with APS-related renal infarction (7–28). The median age at renal infarction was in the 30s (**Figure 3**), and there was a slight female predominance (approximately 55% female). Nearly 30% of the cases represented secondary APS (associated with SLE or other autoimmune disease), while the majority were primary APS. Despite APS being more common in women, several of the renal infarction cases occurred in young men (8, 11, 16, 21, 26, 28). Renal involvement ranged from unilateral to bilateral infarction; bilateral events were associated with worse renal outcomes and sometimes dialysis dependence. In a few reports, renal infarcts occurred after hydroxychloroquine withdrawal in an SLE patient (suggesting HCQ’s protective effect) (17) or in the context of trauma (26) or hormonal therapy (e.g., oral contraceptive pills), where physical or estrogen-related hypercoagulability may have precipitated APS thrombosis (25). Cases have also been described after blunt trauma, adenomyosis treatment with dienogest, and in association with other triggers in young individuals (16, 20). These cases collectively illustrate that APS-related renal infarction, while rare, can occur in young individuals and sometimes under unusual circumstances.

**Table 2:** Clinical features of Antiphospholipid Syndrome APS cases with renal infarction: literature review and our case.

First Author (Year)/Ref.	Sex	Age	Main Clinical Presentation	Renal Infarct Laterality	Pathological Findings	aPL Antibody Profile	Anticoagulant Treatment	Comorbid Conditions	Outcome/Prognosis
Pérez et al. (1992)/7	M	57	Fulminant multiorgan arterial/venous thromboses (acute multiple organ failure)	Bilateral (both kidneys involved)	Thromboses with ischemic necrosis in multiple organs on autopsy	LA+; aCL-IgG+; false-positive VDRL	None (diagnosis made post-mortem)	None (Primary APS)	Fatal (died of widespread thromboses)
Ames et al. (1992)/8	M	43	Malignant hypertension and acute oliguria (renal failure)	Bilateral (occlusion of both renal arteries)	Renal vessel biopsy: immune-complex vasculitis in arterial walls	aPL+ (type NR); abnormal protein C/S levels	heparin then warfarin initiated	None (Primary APS)	Survived (persistent renal impairment; required management)
Sonpal et al. (1993)/9	F	22	Recurrent stroke (CVA), renovascular hypertension, and flank pain	Unilateral (left renal infarct)	Angiogram: no perfusion to left lower pole; elevated renal vein renin	aCL + (high titers)	heparin & warfarin started	None (Primary APS)	Survived (managed with anticoagulation and BP control)
Perinbasekar et al. (1995)/10	F	66	Recurrent flank pain episodes; mild renal insufficiency	Bilateral (recurrent multiple infarcts)	None reported (diagnosis by imaging)	aCL-IgG+	Low-dose aspirin therapy only (no warfarin needed)	Mixed Connective Tissue Disease (MCTD)	Recovered fully (lesions resolved; normal renal function in 3 weeks)
Poux et al. (1996)/11	M	35	Acute flank pain with severe hypertension; aortic occlusion on imaging	Unilateral (renal infarction of right kidney)	Thrombosis of infrarenal aorta with renal infarct on imaging	LA+	IV heparin → long-term warfarin	None (Primary APS)	Survived (managed with warfarin; stabilized renal function)
Hernández et al. (1996)/12	F	*25*	Malignant hypertension; acute flank pain (renal infarct) in young woman	Unilateral (single kidney infarct)	Renal artery thrombosis confirmed by arteriography; renal scan showing infarct	aPL + (type NR)	Warfarin not reported (patient was on ASA, steroids, etc.)	SLE diagnosed 14 years later	Deteriorated (multiple strokes over years; progressed to death despite therapy)
Sá et al. (1999)/13	F	*NR*	Acute unilateral flank pain; nephrotic-range proteinuria (nephrotic syndrome)	Unilateral (total infarction of right kidney)	*NR* (presumed APS nephropathy on biopsy – not reported)	aPL+ (type NR)	IV heparin → oral anticoagulant (warfarin)	None (Primary APS)	Survived (complete infarction of right kidney)
Moriuchi et al. (1999)/14	F	20	SLE flare with severe hypertension and abdominal pain (renal infarctions)	Bilateral (multiple renal infarcts)	diagnosis by imaging; no pathology reported	LA+	Heparin → warfarin	SLE (secondary APS)	Survived (managed with anticoagulation and immunosuppression)
Kitta et al. (2001)/15	M	51	Sudden right flank pain (acute right renal artery thrombosis); recurrent left flank pain 2 months later	Bilateral (asynchronous RIs – right then left)	No kidney biopsy; imaging showed occluded renal arteries; LV thrombus noted on echocardiography	LA+; aCL+; β2GPI+	Thrombolysis (local urokinase) acutely; long-term warfarin	Hypertrophic cardiomyopathy (predisposed LV thrombus)	Alive and stable (no new thromboses at 6-month follow-up on warfarin + antiplatelet)
Huang et al. (2009)/16	M	34	Migratory abdominal pain → severe bilateral flank pain; acute kidney injury & new-onset severe hypertension	Bilateral	Renal biopsy: focal segmental glomerulosclerosis (no vasculitis)	LA+	Enoxaparin (LMWH)	Polyarteritis nodosa (PAN) coexisting with APS	Recovered – renal function normalized; hypertension resolved.
Zenone et al. (2011)/17	F	34	Acute abdominal pain with fever and transient ileus; BP 160/100. After stopping hydroxychloroquine	Bilateral	Renal biopsy not done	LA+; aCL+; β2GPI+	Heparin → Warfarin (INR 3–4)	SLE (10-year history); obstetric APS (recurrent miscarriages, eclampsia)	Favorable – stabilized on anticoagulation; no further thromboses reported.
Hong et al. (2012)/18	F	14	SLE flare: 2 days of vomiting & diffuse recurrent abdominal pain (lupus mesenteric vasculitis)	Left	Renal biopsy not done	aCL-IgG+; LA+)	Enoxaparin → Warfarin	SLE (1-year history, poorly compliant)	Recovered – no recurrence of vasculitis or infarction at 10 months on warfarin + low-dose pred/HCQ.
Padilla-Fernández et al. (2013)/19	F	69	Left flank pain with vomiting	Bilateral (multiple segmental infarctions)	No biopsy; CT and Doppler showed wedge-shaped renal infarcts	aCL-IgM+	IV heparin infusion; discharge on LMWH	SLE (recently diagnosed; on acenocoumarol for atrial fibrillation)	Improved (symptoms resolved; no new lesions on repeat CT; preserved renal function)
Scully et al. (2013)/20	M	50	Recurrent embolic renal infarctions: left native kidney and later transplanted kidney due to LV mural thrombi	Bilateral (native & transplant)	NR	β2-GPI-IgM+	Heparin → Warfarin (lifelong)	Renal cell carcinoma; diabetes mellitus; prior MI with LV aneurysm (mural clot)	Managed with life-long warfarin; no further infarcts after APS confirmed
Zhou et al. (2016)/21	M	43	Recurrent flank/abdominal pain episodes over 2 years; initial misdiagnosis (appendectomy)	Bilateral (heterochronic RIs – right then left)	No biopsy at infarct; imaging showed multiple wedge infarcts; genetic testing revealed MTHFR & PLG mutations	LA+	Acute: heparin; Chronic: lifelong warfarin (INR 2–3)	None (Primary APS with thrombophilia)	Improved (partial renal function recovery; stable on warfarin)
Ugan et al. (2016)/22	F	36	Ischemic left hand and new-onset severe hypertension; then acute left abdominal/flank pain with rebound tenderness	Left	NR	β2-GPI+	Thrombolysis (rtPA) + Heparin; maintenance Warfarin	None (primary APS)	Recovered – BP normalized, partial recanalization of left renal artery.
Nagata et al. (2017)/23	F	48	Incidental mild proteinuria for 3 years; then acute right back/flank pain (renal infarction)	right renal infarct; left renal artery stenosis	Prior renal biopsy showed arteriolar fibrous intimal hyperplasia & APS microangiopathy	LA+; aCL+; anti-β2-GPI+ (triple-positive APS)	Heparin followed by warfarin (anticoagulation only)	None (Primary APS)	Good (stabilized on anticoagulation; no other thromboses)
Kuchera et al. (2021)/24	F	59	12 h of sharp left upper & lower quadrant pain with nausea/vomiting	Left	NR	LA+; aCL+; anti-β3-GPI+ (triple-positive APS)	IV Heparin infusion → Warfarin (lifelong)	Severe centrilobular emphysema (COPD) with cor pulmonale; biventricular heart failure	Improved – creatinine fell from 2.34 to 1.71; discharged on lifelong warfarin
Cross et al. (2022)/25	F	26	Right lower quadrant abdominal pain (initially mimicking appendicitis)	Right kidney	No biopsy (diagnosis by contrast CT)	aCL+	Heparin → oral rivaroxaban anticoagulation	None (Primary APS)	Good (recovery with anticoagulation; no recurrence)
Lee *N.A.* et al. (2022)/26	M	20	Abdominal trauma (sled accident) with acute left flank pain	Unilateral (left kidney; global infarction)	No biopsy (angiography attempted revascularization; confirmed total occlusion)	LA+	Enoxaparin → warfarin (lifelong anticoagulation planned)	None (Primary APS)	Good (LA confirmed at 12 weeks; stable on warfarin; left kidney atrophic, renal function normal)
Lee *K.-Y.* et al. (2025)/27	F	48	Severe abdominal pain 1 month after starting dienogest for adenomyosis	Bilateral (infarctions in both kidneys)	No biopsy (contrast CT: bilateral renal infarcts; mural aortic thrombus)	aPL+(type NR)	IV heparin → oral edoxaban (factor Xa inhibitor)	Adenomyosis (on progestin therapy)	Good (recovered under anticoagulation; no recurrence over 2-year follow-up)
Cid Puente et al. (2025)/28	M	39	Left leg DVT → rapid CAPS: multi-organ infarcts (lungs, liver, spleen, left kidney)	left kidney infarct among multi-organ	No biopsy (clinical CAPS; infarcts confirmed on imaging)	LA+	High-dose IV heparin infusion; plus therapeutic warfarin initiated	None (Primary APS)	Fatal (CAPS progression despite treatment; multi-organ failure within 3 days)
present Case	M	23	14 days of left flank pain with fever, nausea/vomiting;	Unilateral (left kidney; global infarction)	Percutaneous renal biopsy (day 19): diffuse renal cortical necrosis consistent with ischemic infarction	aCL-IgG+; LA+	LMWH (enoxaparin) bridge → warfarin, target INR 2.5–3.0, long-term	Primary APS; with ds-DNA+, anti-nucleosome+, SSA+, MPO-ANCA+, PR3-ANCA+, and anti-GBM+, does not meet criteria for SLE, AAV, or ant-GBM disease	Fever and pain resolved; no recurrent thrombosis >2 years follow-up; proteinuria negative; preserved renal function; left renal scar with right kidney compensation

### Clinical presentation and diagnosis

The presenting symptoms of renal infarction are nonspecific: **f**lank or abdominal pain, fever, nausea, hematuria, and sometimes hypertension (8, 9, 11, 12, 14, 16, 22). Our patient presented with flank pain, fever, and hematuria, all common but nonspecific clues. Elevated LDH, hematuria, and modest renal dysfunction are often reported. In our case, LDH was modestly elevated, and creatinine was only slightly increased, likely reflecting unilateral involvement with preserved contralateral kidney. Diagnosis relies on a high index of suspicion and appropriate imaging. Contrast-enhanced CT or CT angiography is the imaging modality of choice; in our patient, CTA elegantly showed a segmental left renal artery defect and extensive cortical non-enhancement. The diagnosis of APS in this setting requires documented arterial thrombosis plus persistent aPL on two occasions ≥12 weeks apart. Our patient fulfilled these criteria: he had a documented arterial thrombosis (renal artery infarction) and persistent LA and high-titer aCL-IgG on repeat testing, with no evidence of other systemic autoimmune disease, supporting a diagnosis of primary APS.

### Role of complement (C4d) in APS thrombosis

A notable finding in this case was the strong C4d staining along glomerular capillary and small arterial walls in the infarcted kidney. C4d is a byproduct of the classic complement pathway often used as an immunohistochemical marker of antibody-mediated tissue injury, particularly in transplant pathology where diffuse peritubular capillary C4d deposition indicates antibody-mediated rejection. Its presence in a native kidney in an APS case is significant. Increasing evidence suggests that complement activation is involved in APS pathogenesis. Antiphospholipid antibodies can activate complement on endothelial surfaces, promoting endothelial injury and thrombosis (29). In APS-associated thrombotic microangiopathy, strong C4d and C5b-9 deposition has been observed in kidney biopsies (30), including in catastrophic APS or APS nephropathy where renal biopsies showed C4d in vessels, similar to our case. Our findings reinforce that APS can cause complement-mediated endothelial injury and thrombosis in renal vasculature, without immune-complex glomerulonephritis. Importantly, there was no glomerular proliferative lesion or immune-complex deposition by immunofluorescence, favoring a thrombotic rather than immune-complex GN mechanism. This aligns with experimental and clinical data that have linked complement activation to fetal loss and thrombosis in APS and provides a rationale for exploring complement inhibition in addition to standard anticoagulation in selected refractory APS cases.

### Differential diagnosis considerations

Our patient’s course highlights the complexity of differential diagnosis in a young man with renal infarction and multiple transient autoantibody positivities. Initially, we saw positive dsDNA and nucleosome antibodies, which raised concern for SLE. However, these were likely false positives. Transient autoantibody elevations can occur in acute inflammatory states and in APS itself. Anticardiolipin antibodies, for example, are known to cause a false-positive VDRL (syphilis test) by cross-reactivity, and one could speculate that polyclonal B-cell activation in APS or infection may transiently raise various autoantibody titers without indicating true SLE or vasculitis. Repeat testing in our patient showed that dsDNA, nucleosome antibodies, ANCA, and anti-GBM all reverted to negative, and ANA remained negative, while LA and aCL-IgG persisted, strongly arguing against SLE, ANCA-associated vasculitis, or anti-GBM disease.

ANCA-associated vasculitis can present with renal involvement and positive ANCA serology, but typical presentations include rapidly progressive glomerulonephritis with crescents and often pulmonary involvement (31). Biopsy in our patient showed no crescents and no immune-complex or pauci-immune GN pattern; instead, there was pure cortical necrosis with thrombotic occlusion of small arteries, arguing against AAV. Anti-GBM disease can also present with positive anti-GBM antibodies, but those patients usually present with severe glomerulonephritis or lung hemorrhage and show linear IgG deposition along glomerular basement membranes, which was absent in our patient (32–34).

Other differentials include SLE-associated renal infarction and polyarteritis nodosa (PAN). In young patients with renal infarction, SLE can cause a similar presentation, but SLE-related vasculitis or thrombotic lesions typically occur in the setting of systemic features (rash, cytopenias, serositis) and significant immune-complex deposition on biopsy (which was absent in our case). PAN is another differential for multiple intra-renal or visceral arterial aneurysms and infarcts, but our patient had none of the systemic features of PAN (no aneurysms on angiography, no mononeuritis multiplex, no gastrointestinal ischemia, no systemic medium-vessel involvement). Thus, careful integration of clinical, serologic, and pathologic data is required to avoid misdiagnosis. Clinicians should be aware of these potential red herrings. We have flagged the phenomenon of spurious autoantibody positivity as an important learning point from this case.

### Management of APS-related renal infarction

The cornerstone of treatment for APS-related arterial thrombosis is prompt and sustained anticoagulation, typically with a vitamin K antagonist (warfarin) targeting an INR of 2.0–3.0 or 2.5–3.5 in high-risk arterial APS. In our patient, early LMWH followed by warfarin likely contributed to good outcomes with preserved overall renal function. The role of adjunctive therapies (aspirin, hydroxychloroquine, and immunosuppression) in primary APS without SLE remains debated. Hydroxychloroquine has endothelial-protective and anti-thrombotic effects and is standard in SLE; observational data suggest it may reduce thrombosis risk in APS as well. Low-dose aspirin is often used in arterial APS or high-risk aPL profiles; we opted for combination therapy (warfarin plus low-dose aspirin plus hydroxychloroquine) given the severity of the arterial event and multi-positive aPL profile.

Direct oral anticoagulants (DOACs) remain controversial in APS, especially in patients with arterial events or triple aPL positivity, where increased risk of recurrent thrombosis has been reported (25, 27). Current guidelines generally discourage DOACs in high-risk APS, favoring vitamin K antagonists. Our patient, with an arterial event and persistent LA and high-titer aCL-IgG, was maintained on warfarin rather than switched to a DOAC (15, 17, 23, 24).

A subset of APS patients with renal involvement might have catastrophic APS or APS-associated thrombotic microangiopathy, where more aggressive treatment (high-dose steroids, plasma exchange, IVIG, and sometimes rituximab or complement inhibitors) is indicated (28). In our case, there were no features of catastrophic APS (no multi-organ failure), and the patient responded well to anticoagulation and a moderate glucocorticoid taper, so escalation to such therapies was not required.

### Prognosis

The prognosis of APS-related renal infarction is variable and depends on laterality, extent of infarction, time to diagnosis, and adequacy of anticoagulation. In our pooled review, many patients with unilateral infarction preserved global renal function on follow-up, although a few were left with chronic kidney disease (especially those with bilateral infarctions or delayed diagnosis). Early recognition and treatment appear critical for kidney salvage. Long-term management also requires attention to cardiovascular risk factors and avoidance of additional pro-thrombotic triggers (e.g., smoking, estrogen-containing contraceptives) as an important aspect of management.

## Summary

In summary, we report a rare case of primary APS presenting as a unilateral renal infarction with cortical necrosis in a young man, with biopsy-proven thrombotic cortical necrosis and C4d deposition in the kidney. The case emphasizes that APS should be considered in young patients with unexplained renal infarction, that transient positivity of multiple autoantibodies can be misleading and requires repeat testing, and that complement activation may play a pivotal role in APS-related renal thrombosis. Prompt anticoagulation and appropriate adjunctive therapy, along with careful long-term follow-up, can lead to excellent outcomes, as seen in our patient.

## Data Availability

The original contributions presented in the study are included in the article/supplementary material. Further inquiries can be directed to the corresponding author.
